# Renal Cases: A Series of Selected Clinical Reports and Surgical Studies

**Published:** 1900-12

**Authors:** 


**Renal Cases: a Series of Selected Clinical Reports and Surgical Studies.** By David Newman, M.D. Pp. xii., 151. Glasgow: James MacLehose & Sons. 1899.

No one could carefully read these records, and the comments of the author upon them, without gaining considerable knowledge, and that of a kind likely to prove specially useful in the diagnosis of obscure cases of renal disorder. It is shown that renal pain and colic, and intermittent hæmaturia, albuminuria, and hydronephrosis, may be cured by nephrorraphy. The pathology and symptomatology of cystic disease of the kidney is more fully discussed than in ordinary text-books of surgery, and even more fully than in many special works. The differential diagnosis of the various causes of hæmaturia is also fully discussed. The special lessons to be learnt from each case recorded are clearly expressed. Many details are given in these records which, though they yielded valuable help in diagnosis, would in practice have easily escaped the attention of any but the most careful observers. The cases seem to well support the conclusions and opinions of the author.

